# Chromatin as a Platform for Modulating the Replication Stress Response

**DOI:** 10.3390/genes9120622

**Published:** 2018-12-11

**Authors:** Louis-Alexandre Fournier, Arun Kumar, Peter C. Stirling

**Affiliations:** 1 Terry Fox Laboratory, BC Cancer Agency, Vancouver, BC V5Z 1L3, Canada; lfournier@bccrc.ca (L.-A.F.); akumar@bccrc.ca (A.K.); 2 Department of Medical Genetics, University of British Columbia, Vancouver, BC V5Z 1L3, Canada

**Keywords:** chromatin, replication stress, histone chaperone, chromatin remodeller, genome instability

## Abstract

Eukaryotic DNA replication occurs in the context of chromatin. Recent years have seen major advances in our understanding of histone supply, histone recycling and nascent histone incorporation during replication. Furthermore, much is now known about the roles of histone remodellers and post-translational modifications in replication. It has also become clear that nucleosome dynamics during replication play critical roles in genome maintenance and that chromatin modifiers are important for preventing DNA replication stress. An understanding of how cells deploy specific nucleosome modifiers, chaperones and remodellers directly at sites of replication fork stalling has been building more slowly. Here we will specifically discuss recent advances in understanding how chromatin composition contribute to replication fork stability and restart.

## 1. Replication of Chromatin

Nucleosomes are the basis of chromatin organization in eukaryotes. Nucleosomes are assembled by the sequential association of two histone H3–H4 dimers and two histone H2A–H2B to form an octameric protein complex that wraps about 147 base-pairs of DNA [1]. Nucleosome assembly requires the aid of protein chaperones that function in the storage, transport, modification or deposition of histones onto DNA [2]. Packaging of DNA with nucleosomes to form chromatin is essential for genome integrity. Histone modifications and dynamics regulate not only chromatin compaction and gene expression but also DNA repair, DNA replication and mitosis [3,4]. Perturbation of any of these processes can lead to DNA damage and mutations.

**Figure 1:**
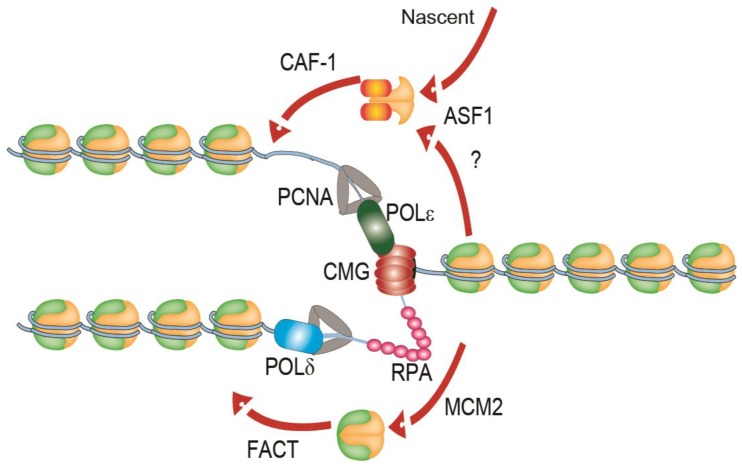
A simplified illustration of a replisome on replicating chromatin. The leading strand polymerase POLε associated with the replicative helicase complex (CMG—Cdc45–MCM–GINS) and an RPA-coated lagging strand is shown. The action of ASF1 and CAF-1 in histone deposition, of nascent histone H3–H4 complexes and potentially recycled histones is shown. The histone recycling function of MCM2 and the FACT (Facilitates Chromatin Transcription) complex are shown below. See main text for additional details. CAF-1: Chromatin_assembly factor 1; ASF1: anti-silencing factor 1; PCNA: proliferating cell nuclear antigen; MCM2: minichromosome maintenance 2; POL: DNA polymerase; RPA: replication protein A.

The process of chromatin replication presents additional challenges to cells. Replication forks themselves are highly complex structures, consisting of a multimeric helicase complex termed CMG (Cdc45–MCM–GINS), multi-subunit leading and lagging strand polymerases, a processivity factor called PCNA (proliferating cell nuclear antigen), a trimer single-stranded DNA (ssDNA) binding protein RPA (Replication Protein A) and numerous other factors (Figure 1). Nucleosomes must be removed ahead of advancing replication forks and be recycled behind the forks onto the nascent sister chromatids to preserve the epigenetic state of the replicated locus (Figure 1). This process is not completely understood and has been reviewed elsewhere [5,6]. Briefly, during replication, newly-synthesized nucleosome core particles histone H3 and H4 can be bound by the histone chaperone anti-silencing function 1 (ASF1), which associates ahead of the replication machinery with the MCM2 (MiniChromosome Maintenance 2) subunit of the core helicase complex [7,8]. ASF1 may also coordinate the first steps of histone recycling at the leading edge through the deposition of histone “pre-disposition marks,” which signals for the disassembled and nascent histone complexes to be re-incorporated into the newly synthesized DNA by other histone chaperones, like the chromatin assembly factor 1 (CAF-1) [9]. CAF-1 is recruited to sites of DNA synthesis through interactions with PCNA [10]. Histone pre-disposition marks include the di-acetylation of H4 at lysines 5 and 12 (H4K5K12) by the H4K5K12 lysine acetyltransferase RBAP46/HAT1 in human cells [11] and the acetylation of H3K56ac and H3K27ac by Rtt109 in budding yeast (H3K14ac and H3K18ac in humans) prior to deposition into newly synthesized DNA [7,11].

While ASF1-CAF-1 driven recycling of H3–H4 is important for replication progression [8], more recent studies reveal crucial intrinsic roles for the replication machinery itself as a histone chaperone. Core subunits of the leading strand polymerase (Polε), POLE3-POLE4, have been demonstrated to have histone chaperone activity and promote histone recycling [12]. The ssDNA-binding protein complex RPA is also present at every replisome and has been ascribed a role in histone deposition. RPA binds directly to H3–H4 complexes and facilitates their deposition into adjacent double stranded DNA, likely in concert with histone chaperones in the cell [13]. Finally, the MCM2 subunit of the replicative helicase also binds directly to histones and works with the FACT complex to promote histone recycling during replication [14]. More recently, the histone recycling function of MCM2 was shown to be essential to maintain epigenetic inheritance of histone post-translational modifications [15]. Thus, the FACT-MCM2 pathway may provide most of the essential histone recycling activity in normal cells. Moreover, the action of various chromatin remodelling complexes such as SWI/SNF (Switching deficient/Sucrose-Non-Fermenting), Imitation Switch (ISWI) and INO80 (Inositol requiring 80) is important for normal replication [16,17]. Thus core replisome components like Polε, MCM2 and RPA, along with regulation by dedicated histone chaperones and chromatin remodellers together ensure histone deposition and recycling in replication, which in turn ensures epigenetic stability during cell division.

## 2. Replication Stress and Fork Stalling

The progression of replication forks can be impaired by various physical barriers. Such replication barriers include DNA–protein and DNA–DNA crosslinks, DNA lesions such as thymidine dimers, DNA secondary structures such as G-quadruplexes, transcription-replication conflicts associated with DNA:RNA hybrid structures called R-loops, topological stresses, depletion of nucleotide pools and other conditions [18]. When replication fork slowing and stalling occur frequently during S-phase, cells experience replication stress. Replication stress notably occurs in cancer where it is likely to drive mutational processes that underlie genome instability phenotypes associated with the disease [19]. Indeed, oncogene activation is sufficient to induce DNA replication stress [20,21].

Stalled replication forks must be restarted or rescued by a converging fork in order to prevent under-replicated DNA entering mitosis. Stalled forks first expose ssDNA that is coated with RPA. RPA stimulates the activation of the Ataxia-telangiectasia and Rad3 related protein (ATR) kinase which initiates a cascade of signalling events that can stall the cell cycle and recruit various effectors to the stalled fork [18]. Given the central role of ATR in responding to replication stress and the prevalence of replication stress in cancer, ATR inhibitors are now under investigation as anti-cancer therapeutics.

Replication stress is often studied by examining specific signalling events, such as phosphorylation of RPA2 on serine 33 (RPA2ser33p), or by directly measuring ssDNA exposure with BrdU incorporation and detection under non-denaturing conditions (where only ssDNA with BrdU binds the antibody). Replication stress is often induced in vitro by damaging DNA with alkylating agents like methyl methanesulfonate (MMS), by creating protein–DNA crosslinks with the topoisomerase I inhibitor camptothecin (CPT), by depleting nucleotide pools with hydroxyurea (HU), or by directly inhibiting DNA replication with aphidicolin. The identity of proteins associated with nascent DNA through the mass-spectrometry based isolation of proteins on nascent DNA (iPOND) approach has helped to characterize the composition of stressed replication forks [22]. Finally, as a gold standard method, the incorporation of halogenated nucleotides during replication enables direct measurements of replisome dynamics on stretched DNA fibres [18]. Together these approaches have dramatically improved our understanding of the composition, signalling responses and impact on replisome dynamics of various types of replication stress.

Depending on the nature and timing of the fork stalling event, replication forks can be restarted via lesion bypass mechanisms such as translesion synthesis, repriming of replication after the lesion, or template switching [18]. Alternatively, the fork can be cleaved to create a broken end that can lead to fork restart by homologous recombination (HR) [23]. Cellular resilience to fork stalling is a complex process that remains an active area of investigation and has been extensively reviewed elsewhere [18]. What may be underappreciated is that, regardless of the restart pathway chosen, the DNA transactions that take place at stalled forks occur in the context of chromatin and relatively little is known about how histone states may serve as scaffolds for, or impediments to, fork restart and cellular recovery. Here we will concern ourselves with specific recent examples of chromatin regulators whose disruption leads to replication stress and to direct effectors of histones that are activated in *cis* to replication fork stalling.

## 3. Histone Modifications in Replication Stress Responses

Dynamic histone modifications and variants are well-established players in the DNA double strand break (DSB) repair response. For example, histone variant H2A.X phosphorylation at breaks is a crucial signalling event to initiate DNA repair reactions [24]. Core histone ubiquitin signalling through the action of RNF8 and RNF168 E3 ligases is now appreciated as an essential platform for organizing HR [25]. The modification of chromatin during replication stress is at least as complex and less understood. Below we illustrate more recent links of dynamic modifications of nucleosomes during replication stress.

### 3.1. H2A Ubiquitination

Ubiquitination of H2A is associated with transcriptional repression [26,27,28]. Evidence of H2A-ubiquitin function in replication stress comes from studies of RING1A and RING1B, two members of the polycomb repressive complex 1 (PRC1) E3 ligase family which ubiquitinate lysine 119 of H2A (H2AK119Ub) [29]. This modification at pericentromeric heterochromatic regions was found to promote S phase progression. Moreover, ring1A and ring1B mutant cells in unperturbed conditions show increased levels of stalled replication forks and DSBs as measured by RPA, γH2A.X and 53BP1 foci [29]. Targeted mono-ubiquitination of H2A (H2AK119Ub) at pericentromeric domains rescues the mutants and re-establishes S phase progression, suggesting that RING1A and RING1B have important roles in modulating H2AK119Ub levels in order to promote genome stability during DNA replication.

Another ubiquitin E3 ligase that targets H2A under conditions of replication stress is RNF168. Loss of H2A ubiquitination by RNF168 was recently found to cause reduction in replication fork progression and reversed fork accumulation [30]. In fact, untreated RNF168-deficient cells display higher levels of fork reversal. Delayed fork progression can be restored upon deletion of factors promoting fork reversal (such as RAD51) suggesting a role for RNF168 in regulating reversed replication fork restart. For instance, inhibition of MRE11 by mirin in the RNF168 mutants rescues the delayed fork progression phenotype to wild type levels. This indicates that RNF168 aids in reversed fork restart and prevents degradation of the reversed fork by monoubiquitinating H2A at K15 (H2AK15Ub) [30]. These data suggest that reversed forks may be recognized by the DNA DSB repair machinery and processed in a similar way, requiring H2A ubiquitination, to promote genome stability at stalled forks.

### 3.2. H2B Ubiquitination

H2B has various functions, including export of messenger RNA (mRNA) [31], 3′-end mRNA processing [32], exon-intron marks [33] and regulation of RNA polymerase II elongation [34,35]. H2B also undergoes mono-ubiquitination at K123 (H2BK123ub) via the action of the Bre1/Rad6 E3 ligase complex [36]. Bre1 has an established function in transcriptional elongation and, accordingly, H2BK123Ub marks are enriched over transcribed regions [37]. Whether H2B ubiquitination occurs dynamically in response to replication stress is unclear. It is clear that loss of normal H2B ubiquitination leads to replication stress. Knockdown of Bre1, or expression of an H2B-K123R mutant that cannot be ubiquitinated have been shown to lead to defective replication fork progression and replication stress [37,38]. This phenotype is attributed to nucleosome stability and assembly behind the advancing replication fork. In fact, H2BUb promotes nucleosome stability during replication by affecting H3K56ac and H3K27me3 histone mark deposition. Impaired H2B ubiquitination has been shown to result in decreased occupancy of H3K56ac and H3K27me3 at origins of replication, suggesting that H2Bub might be involved in the ASF1/Caf1/Rtt109 histone recycling pathway [39]. Another possible source of replication stress in Bre1-deficient cells is R-loops. Bre1-depleted cells show an upregulation of RNA processing genes and accumulate DNA damage. Upon overexpression of RnaseH1, an enzyme which degrades R-loops, DSB levels return to normal, suggesting that R-loops are the cause of replication stress and DNA damage in these mutants [37]. While Bre1 has multiple functions, the phenotypes described above are associated with a reduction in ubiquitination levels of H2B, highlighting a role for this histone mark in the context of replication stress.

When studying DNA damage tolerance pathway activation at stalled forks, a recent study provided evidence that H2BK123ub is required by the replication fork to bypass lesions [40]. *H2BK123R* mutants treated with MMS accumulate replication intermediates at origins, delay origin firing and accumulate higher levels of RPA foci compared to wild type cells, suggesting that H2Bub is important for fork stability in the presence of DNA damage [39,40]. Removal of MMS showed a persistent level of RPA foci indicating that depletion of H2BK123ub affects the repair of replication-associated DNA damage intermediates. In accordance, a role for H2Bub in RAD51-dependent DNA repair was identified. Additional studies of DNA damage tolerance pathways have uncovered that H2Bub aids both Rad18-independent HR and Rad18-dependent translesion synthesis for lesion bypass before and after genome replication [40].

While ubiquitination of both H2A and H2B has been implicated in replication stress, another study suggests that the levels of ubiquitinated H2A and H2B must be tightly regulated for genome maintenance. Loss of USP3, which is a de-ubiquitinase for H2A and H2B, also leads to DNA damage checkpoint activation and delayed S-phase progression [41]. One possible explanation for this phenotype is that the absence of USP3 could lead to hyper-ubiquitination of H2A and H2B, resulting in a sensitized DNA damage response which eventually leads to genome instability. Ubiquitination of H2A and H2B also affect transcription, which could provide an alternative explanation for this phenotype, though this hypothesis remains to be tested experimentally. Ultimately, there is an increasing body of evidence suggesting a role for the ubiquitination of histone H2A and H2B variants in the mitigation of replication stress.

### 3.3. EZH2 and H3K27me3

Histone marks cross-talk with one another through the action of combinatorial histone modification readers, writers and erasers [42]. For example, PRC1 mediated mono-ubiquitination of H2A at lysine 119 (H2AK119Ub) promotes PRC2 binding and the eventual deposition of the H3K27me3 mark on ubiquitin-modified H2A nucleosomes [43]. This modification is catalysed by the PRC2 subunit enhancer of zeste homolog 2 (EZH2), which is a histone methyltransferase that mediates histone H3 lysine 27 di- and tri-methylation (H3K27me2/me3). The H3K27me3 mark is a well-established repressive mark involved in transcription. Recently, a direct role for H3K27me3 at stressed replication forks was proposed. This study found that the recruitment of the MUS81 nuclease to stalled forks was regulated by dynamic increases in local H3K27me3 [44]. The endonuclease activity of MUS81 is involved in resolving recombination intermediates during DSB repair [45]. However, MUS81 recruitment at stalled replication forks promotes cleavage of the fork to promote replication restart [44]. Cells treated with the replication fork stalling agent hydroxyurea (HU) show increased EZH2 and H3K27me3 at stalled replication forks, as shown by co-localization with the replication stress marker RPA2ser33p [44]. The histone methyltransferase activity of EZH2 is required for fork degradation and restart in BRCA2-deficient cells, whereas EZH2 deficiency prevents MUS81 recruitment and enhances stalled replication fork protection [44]. Although MUS81 recruitment at stalled forks is necessary for replication fork restart in BRCA2-deficient cells, it has been proposed that aberrant activity of MUS81 at stalled forks may be detrimental by promoting uncontrolled fork degradation or collapse [44]. Ultimately, this work defines a previously unappreciated regulatory step for MUS81 recruitment to stalled forks involving histone methylation.

### 3.4. SETD1A, MLL3/4 and H3K4 Methylation

SET domain containing 1A (SETD1A) is a histone methyltransferase that modifies histone H3 at lysine 4 (H3K4me1) [46]. SETD1A mediated methylation of H3K4 at stalled replication forks directs the recruitment of the Fanconi anaemia protein FANCD2 to enhance its histone chaperone activity. The mobilization of histones onto the regressed arm of a reversed replication by FANCD2 functions in stabilizing the RAD51 nucleofilament, preventing fork collapse [46]. The exact mechanism of this chaperone activity of FANCD2 at stalled forks is unknown but two hypotheses have been proposed: (1) H3K4 methylation by SETD1A may control the accessibility of chromatin to FANCD2 or (2) H3K4 methylation may promote the recruitment of co-factors that promote nucleosome remodelling associated with FANCD2 [46]. In fact, disruption of SETD1A or the inability to deposit the H3K4me1 histone mark prevents FANCD2-mediated chaperone activity at stalled replication forks and promotes aberrant fork processing, resulting in genome instability [46]. The SETD1A-FANCD2 activity at stalled replication forks is therefore crucial to the ability of a cell to prevent replication-associated DNA lesions. Ultimately, the role of SETD1A in replication stress tolerance highlights the requirement for dynamic chromatin remodelling at stalled replication forks to prevent genome instability. A similar process may be conserved in yeast, where the *Set1* methyltransferase catalyses H3K4 methylation in response to replication stress [46,47].

PAX-interacting protein 1 (PTIP) is an adaptor protein involved in the recruitment of histone methyltransferases (e.g., MLL3/4) and potentially histone acetyltransferases to regulate the deposition of histone marks onto chromatin [48]. Though the chromatin remodelling functions of PTIP in gene expression during development have been the focus of multiple studies, more recent research is beginning to appreciate a role for PTIP in the maintenance of genome integrity by HR [49]. PTIP forms a complex with MLL3 and 4 as part of a H3K4 methyltransferase complex [50]. Deletion of PTIP was found to confer protection against MRE11-mediated degradation of stalled replication forks induced by HU in BRCA1/2 deficient cells [51]. A model proposed by Ray Chaudhuri et al. suggests that BRCA2 functions in disengaging MRE11-mediated degradation at stalled forks to promote restart. This is supported by data suggesting that *Brca2*-deficient cells treated with replication fork stalling agents HU or cisplatin present higher levels of chromatin bound MRE11, a nuclease involved in stalled fork degradation [51]. This accumulation of MRE11 at HU-induced stalled replisomes is dependent upon the establishment of H3K4me1/me3 at replication forks by PTIP and MLL3/4 [51,52]. The chromatin modifying role of PTIP appears to play a key role in the mitigation of stalled replication forks. Together, H3 methylation creates multiple inputs to tune responses to stalled forks and regulate the recruitment of replication stress tolerance factors.

In summary, the post-translational modification of histones appears to be a key process in the detection and mitigation replication stress. Histone modifications may also pre-exist at difficult to replicate regions leading to replication stress. For example, H3-Serine10 phosphorylation occurs in chromatin flanking ectopically formed R-loop regions, which are known to interfere with replication forks, leading to downstream genomic instability [53,54]. Our expanding knowledge of the effectors responsible for the deposition and recognition of histone marks has the potential to uncover new genome instability-based therapeutics for the treatment of cancer.

## 4. Histone Variants in Replication Stress Responses

The canonical core histones are important signalling platforms for post-translational modifications during replication stress that coordinate responses to promote genome stability. However, the deposition and removal of variant histones is also important for replication stress tolerance. Here we outline the roles of major, or recently recognized, variant histones at stalled replication forks.

### 4.1. H2A.X

Upon DSB formation H2A.X, a variant of H2A, gets loaded and phosphorylated at serine 139 (H2A.Xser139p) near the break site [55]. This chromatin mark, now called gamma H2A.X (γ-H2A.X) helps recruit proteins and other factors to the site of damage for repair of the lesion. This phosphorylation also occurs upon induction of replication stress and is carried out in an ATR-kinase dependent manner [56]. The γ-H2A.X variant on chromatin in turn stabilizes ATR at stalled forks to help carry out its functions [57]. In ATR deficient cells however, DNA damage signalling is mediated by ATM and DNA-PKcs [58]. The deposition of γ-H2A.X is a rapid response to damage, as initial levels of γ-H2A.X are found to increase within a minute at stalled forks [55]. Chromatin-based studies revealed that, upon damage, the chromatin is opened which promotes phosphorylation of H2A.X by ATR/ATM/DNA-PKcs to signal for the recruitment of repair proteins [59]. The H2A.X histone variant is therefore crucial to the maintenance of genome integrity in response to DNA lesions induced by replication stress.

### 4.2. MACRO-H2A

Another variant of H2A, macroH2A has been implicated in replication stress response and genome stability. MacroH2A1.2 was found to be recruited to stalled forks in both unperturbed cells and to a greater degree when cells are challenged with the DNA polymerase inhibitor aphidicolin [60]. This suggests that macroH2A1.2 is an epigenetic mark present at fragile replication sites in both wild type and stressed cells to help facilitate fork progression. Interestingly, macroH2A1.2 deposition recruited BRCA1 via an interaction with its N-terminal domain at stalled replication forks to possibly help with resection of replication intermediates [60]. MacroH2A1.2 knockdown leads to increased accumulation of γ-H2A.X, RPA and ATM phosphorylation at fragile loci. Furthermore, macroH2A1.2 was deposited onto fragile sites in a FACT-dependent manner. FACT, which has been implicated in R-loop resolution and genome stability [61,62], was found to be a chaperone for deposition of macroH2A1.2. Therefore, the proposed model states that upon replication stress at fragile genomic sites, macroH2A1.2 is recruited in a FACT-dependent manner which in turn recruits BRCA1 to stalled forks to promote fork restart [60].

### 4.3. H2A.Z and Its Regulators

The Ino80 and Swr1 multi-subunit chromatin remodellers are a part of a conserved family responsible for the removal and deposition of the H2A variant H2A.Z [63,64,65]. H2A.Z has functions in genome maintenance, particularly in DSB repair [66]. The potential importance of H2A.Z in replication stress was recently suggested through a study which showed that *HTZ1* (yeast H2A.Z) or *SWR1* deletion in replication fork checkpoint defective strains leads to an increase in gross chromosomal rearrangements (GCRs), replication intermediates and stalled forks, as well as enhanced sensitivity to chemicals that cause replication stress [67]. The authors suggest that transiently stalled forks are protected by the action of Swr1/Htz1 when the replication checkpoint is impaired [67]. INO80-catalyzed removal of Htz1 in yeast also seems to be important for replication stress tolerance as reducing Htz1 expression levels in INO80 mutants rescues some of the replication defects observed in yeast [68]. Thus, it seems that the dynamic deposition and removal of H2A.Z at stalled replication forks are both important for genome maintenance, reminiscent of the function of H2A.Z in DNA repair [63]. INO80 also seems to have functions in DNA replication and replication stress tolerance that may be independent of H2A.Z as described below.

In summary, the cellular response to replication stress requires the incorporation of specific histone variants to promote the maintenance of genome integrity. Described above are only a few examples of the network of chromatin modifying events that occur at stalled replication forks and vary with different chromatin contexts (e.g., heterochromatin, telomeres, centromeres) [29,69,70]. While we will not discuss them at length, it is notable that H3 variants have also been linked to replication stress and DNA damage tolerance. For example, H3.3 is important for replication fork progress after UV damage, although the mechanism is poorly understood [71]. The H3 variant CENP-A needs to be removed by the CHRAC14 chaperone following DNA damage, including replication blocking stresses like MMS treatment [72]. Understanding, the potential interplay of not only H2A but also H3 variants will be important for a complete picture of chromatin-level responses to replication stress.

## 5. Readers, Remodellers and Chaperones in Replication Stress

**Figure 2:**
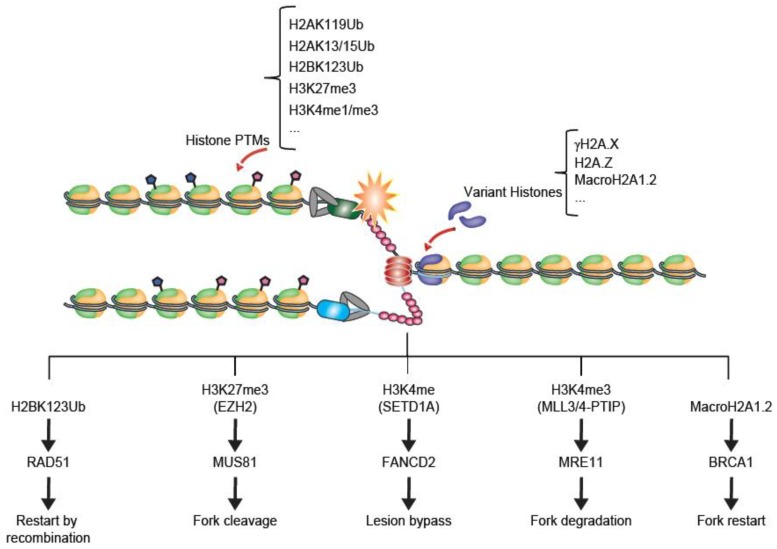
Examples of histone modifications and variants at stalled replication forks. A replication fork stall (orange star) can lead to the exposure of single-stranded DNA (ssDNA) as the helicase separates from the polymerase. This serves as a signal for replication stress which can catalyse local histone modifications or variant incorporation (see main text). Below, five representative outcomes described in this review are shown. In each case, the chromatin-level change is noted at the top and a fork protection and restart protein is recruited to lead to resolution for the stalled fork. In reality these events are certain to be highly coordinated with one another.

As discussed above, histone modifications and variant histones are key early steps in the response of chromatin to replication stress. Indeed, many variants and modified histones play direct roles in recruiting replication stress response factors such as BRCA1, FANCD2, MUS81 or MRE11 which function directly to stabilize and restart stalled replication forks (Figure 2) [44,46,51]. In addition to these response, chromatin remodellers and histone chaperones also mount responses to replication stress that influence genome maintenance and stress tolerance.

### 5.1. INO80

The INO80 complex is recruited to replisomes and stalled forks in both normal and replication stress conditions and is necessary for fork restart and recovery upon removal of replication stress [73]. Accordingly, deletion of INO80 results in stalled forks, suggesting it is involved in fork progression [74]. In yeast, INO80 also modulates ubiquitination of PCNA and influences RAD51-driven processing of stalled forks [75]. In human and mouse cells, INO80 is recruited to normal replisomes by the H2AK119Ub mark and the BAP1 protein that works to stabilize INO80 at these sites [76]. A role for mammalian INO80 in replication stress tolerance is less clear, although INO80 does colocalize with γ-H2A.X at fragile and difficult to replicate genomic regions, which may suggest a function for INO80 in replication fork robustness and recovery similar to the data in yeast [77]. Another observation in the yeast system is that INO80 has a function in removing RNA polymerase complexes that can cause transcription-replication conflicts and replication stress [78]. Whether INO80 plays a direct role in mitigating such conflicts in human cells is currently not known.

### 5.2. TONSL-MMS22L

Tonsoku-like, DNA repair protein (TONSL) recognizes H4 histones unmethylated at lysine 20 (H4K20me0), a mark associated with newly replicated DNA, through its Ankyrin Repeat Domain (ARD) [79]. The H4K20me post-replicative histone mark is believed to function like the H3K56ac mark in budding yeast, in that both identify newly replicated chromatin [79,80]. Association of the TONSL–MMS22L complex to newly replicated chromatin functions in facilitating RAD51 loading at stalled replication forks to promote fork stability and reduce replication-associated DNA damage [79,81]. Importantly, recruitment of TONSL–MMS22L to nascent chromatin increases during replication stress [82]. Evidence suggests that TONSL–MMS22L functions as a histone chaperone and a histone reader, whereby it is loaded onto nascent chromatin containing the H4K20me0 mark during replication via an interaction with the ASF1-MCM2 pre-disposition complex [79]. Accumulation of TONSL–MMS22L onto newly synthesized histones at stalled forks has been suggested to prevent chromatin compaction by sequestering the histone H4 tail from interacting with the H2A–H2B dimers of neighbouring nucleosomes, therefore promoting RAD51 at replication-associated DNA lesions during S-phase [79]. Additionally, TONSL–MMS22L has been demonstrated to preferentially associate with histones H3 mono-methylated at lysine 9 (H3K9me1), a mark enriched on newly synthesized histones that promotes the interaction of H3.1–H4 variants with ASF1 [83]. Accumulation of this nascent histone mark at replication forks correlates with the recruitment of TONSL to promote local chromatin remodelling and inactivation of the replicative helicase through direct binding [83]. In fact, the TONSL–MMS2L complex interacts with the MCM5 subunit of the replicative helicase and may promote nucleosome eviction at the fork [83]. Moreover, through its interaction with MCM5, TONSL is believed to promote replication fork disassembly at stalled forks [83]. This further demonstrates the role of TONSL–MMS22L at the replication fork to promote genomic stability. Disruption of TONSL or MMS22L results in replication-associated DNA damage and replication stress [83,84,85].

### 5.3. SUMO2-Modified PCNA Recruitment of Histone Chaperones

SUMO2 conjugation to lysine 164 of PCNA (K164SUMO2-PCNA) occurs when the replisome collides with the transcription machinery during transcription-replication conflicts [86]. This modification of PCNA is mediated by the RECQ5 helicase that associates with RNAPII during transcription. Although the SUMO-E3 ligase that conjugates SUMO2 to PCNA has yet to be identified, it has been suggested that RECQ5 might recruit PIAS1 to modify PCNA at K164, however the exact mechanism remains to be elucidated [86]. At the conflict, SUMO2-PCNA promotes the enrichment of the histone chaperones CAF-1 and FACT at the stalled replication fork to prevent associated DNA lesions [86]. The recruitment of these histone chaperones is mediated via their SUMO-interacting motifs (SIMs) and promotes the deposition of histone replication-associated H3.1 variants onto chromatin as well as repressive histone marks (i.e., H3K9me2/me3) to reduce chromatin accessibility [86,87]. Additionally, the SUMOylated PCNA–CAF-1 interaction is also believed to enhance CAF-1’s histone deposition activity [86]. Consistent with this model for SUMO2-PCNA function in replication stress tolerance, overexpression of SUMO2-PCNA or CAF-1 can rescue genome instability associated to transcription-replication conflict-prone cells lacking RECQ5 [86]. Moreover, the inability to conjugate SUMO residues onto PCNA at replication forks show an increase accumulation of DSBs [88]. In this way, specific stress-induced modifications of core replisome components like PCNA can ultimately affect the local chromatin state during replication stress.

### 5.4. ASF1-CAF1

Histones H3 deposited by ASF1 onto replicating chromatin are di-acetylated at lysines 5 and 12 (H3K5K12diAc), with about 20–30% of these histones containing tail-acetylation marks at lysines 14 and/or 18 (H3K14K18Ac) [89]. ASF1 is also involved in the methylation of histone H3 lysine 9 (H3K9me1) prior to its deposition into nucleosomes [89]. Evidence suggests that this mark can be deposited by SETDB1 in complex with HP1α and CAF-1, raising the possibility that H3–H4-bound ASF1 docks onto CAF-1 as H3K9me1 gets deposited [89,90]. In addition, a proportion of H3 protein may be modified by SETDB1 co-translationally on polysomes [91]. Under conditions of replication stress induced by HU, ASF1 complexes have been shown to accumulate this mark (H3K9me1) [89]. This data suggests that parental histones being actively shuttled by ASF1 upon fork stalling are maintained in an ASF1-H3-H4-MCM complex on chromatin [89]. Upon resolution of replication stress, such pre-marked histones can be rapidly incorporated into newly synthesized DNA at the risk of potential unscheduled silencing, since H3K9me1 is a precursor to the H3K9me3 heterochromatin-associated mark [89,90]. Ultimately, the function of ASF1 in histone H3–H4 recycling at the replication fork is crucial to the mitigation of replication stress and fork restart. Accordingly, disruption of ASF1 sensitizes cells to replication stress-inducing agents and predisposes cells to replication-associated genome instability [5,89,92]. Moreover, disruption of ASF1 is associated with impaired recycling of parental H3.1 and H3.3 at sites of active replication, which causes changes in histone H3.1 spatial distribution [93]. Much like ASF1, deficiency in CAF-1 has been associated with replication stress. Knockdown of CAF-1 has been shown to slow replication fork progression and activate cell cycle checkpoints [94]. Moreover, cells deficient for CAF-1 have increased serine 317 phosphorylation (S317p) of the checkpoint kinase protein Chk1 by ATR, a PTM associated with replication stress [94].

### 5.5. SWI/SNF and Related Chromatin Remodellers

SWI/SNF is a yeast chromatin remodelling complex family related to human BAF and PBAF which is involved in a variety of cellular processes, including DNA repair and the regulation of transcriptional programs. Evidence suggests that the histone chaperone ASF1, involved in nucleosome remodelling at DNA replication forks, can associate with SWI/SNF and modulate its activity [95]. A study from Minard et al. shows that SWI/SNF and ASF1 work independently to promote rapid de-repression of DNA damage response genes when cells are subjected to replication stress induced by HU or MMS [95]. Accordingly, yeast cells with dual deletions in *ASF1* and *SNF2* present acute cell cycle progression defects in the presence of HU [95]. It has been proposed that this defect is caused by the inability of these mutant cells to proceed into S-phase to activate the checkpoint that triggers the transcription of DDR genes necessary to the repair of HU-induced lesions and progress through the cell cycle [95].

Rsc2 is a component of a yeast SWI/SNF family complex similar to the human PBAF. Deletion of *RSC2* in yeast leads to an acute decrease of PCNA ubiquitination (PCNA-K64ub) under conditions of replication stress induced by MMS treatment [96]. Similar results are observed in human cells when the human ortholog BAF180 is depleted under conditions of replication stress induced by HU or MMS treatment [96]. The depletion of ubiquitinated PCNA in BAF180-depleted cells is caused by a decrease of the chromatin bound E3 Ub-ligase Rad18 and a decrease in chromatin-associated unmodified PCNA [96]. In their model, Nimii et al. propose that the nucleosome remodelling function of PBAF to promote PCNA ubiquitination by Rad18 is required at stalled replication forks to assist repriming of the stalled fork for restart [96]. This does not exclude another mechanism where BAF180 might help the replicative helicase to progress past the lesions responsible for the fork stall [96]. Additional data suggests that BAF/PBAF localizes to replication forks during development via its BRG1catalytic subunit [97]. Another PBAF subunit, PBRM1, has been shown to influence replication stress in VHL-deficient cancer models. Loss of PBRM1 can rescue a VHL-dependent replication stress phenotype by modulating heterochromatin formation via H3K9 tri-methylation (H3K9me3) [98]. Down-regulation of VHL induces replication stress by depleting cellular supplies of RAD51 and RPA, factors involved in stalled replication fork protection and restart [98,99,100]. Loss of PBRM1 under such conditions is believed to alter H3K9me3 marks within the chromatin landscape, which can in turn rescue VHL-dependent replication stress through the modulation of the DDR response [98]. While the details of each story remain to be fully elucidated, together these studies support a role for chromatin-mediated localization and function of the BAF and/or PBAF complexes at sites of normal DNA replication and at stalled replication forks.

### 5.6. ALC1/CHD1L

Amplified liver cancer 1 (*ALC1*—also known as CHD1L) is a chromatin remodelling factor involved in the regulation of replication fork progression and the tolerance of replication stress. ALC1-deficient cells and mutants for the ATPase activity of ALC1 have increased sensitivity to CPT, a TOP1 inhibitor known to induce replication fork slowing and collapse [101]. DNA combing experiments have shown that ALC1 functions in slowing replication fork speed in CPT-treated cells to prevent transcription-coupled genome instability [101]. A study by Ooka et al. suggests that ALC1 remodels chromatin at sites of replication stress to promote the opening of chromatin [101]. Evidence from this article also suggests that ALC1 may collaborate with the PARP pathway to regulate replication fork speed at TOP1-cc lesions, whereby activation of PARP1 by the ATR-Chk1 checkpoint machinery due to stalled forks recruits ALC1 [101]. At sites of stalled forks, ALC1 could remodel the local chromatin environment to facilitate replication fork reversal, allowing for the repair of TOP1-cc lesions [101]. ALC1’s function in DNA replication stress tolerance could explain why it is amplified or overexpressed in a variety of cancers, as this could provide a mechanism for cancer cells to escape otherwise lethal replication-associated DNA lesions [101,102,103].

In summary, replication stress triggers a dynamic chromatin remodelling response that requires cooperation between histone readers, remodellers and chaperones to maintain genome integrity. Uncovering the specific role of the players involved in this complex response is crucial to our ability to understand how cells tolerate replication stress and to improve upon genome instability-based therapeutics.

## 6. Conclusions

Research aimed at understanding regulators of DNA replication stress is growing dramatically. Much of this work has naturally focused on understanding the DNA transactions that promote lesion bypass and fork restart, or fork cleavage. However, replication stress signalling, fork reversal, lesion bypass and fork cleavage all appear to be influenced by changes to chromatin through the action of histone modifying enzymes, histone variants, or chromatin remodellers. Indeed, the integration of histone post-translational modifications, histone variants and the action of histone chaperones and remodellers, must together enable both normal replication and replication stress tolerance in cells. Just as chromatin is recognized as a key regulator of DNA DSB repair, it will be important that models of replication stress tolerance be understood in the context of chromatin. This is because there is great current interest in the anti-cancer therapeutic potential of both replication stress targeting agents such as ATR inhibitors [104] and in epigenetic therapies, which may, for example, inhibit histone methylation or deacetylation [105,106]. The intersection of these fields presents new opportunities to understand a complex biological system and improve human health.
